# Physicochemical, Thermal and Rheological Properties of Pectin Extracted from Sugar Beet Pulp Using Subcritical Water Extraction Process

**DOI:** 10.3390/molecules26051413

**Published:** 2021-03-05

**Authors:** Seyed Hadi Peighambardoust, Maryam Jafarzadeh-Moghaddam, Mirian Pateiro, José M. Lorenzo, Rubén Domínguez

**Affiliations:** 1 Department of Food Science, College of Agriculture, University of Tabriz, Tabriz 5166616471, Iran; mjafarzadehmogaddam@gmail.com; 2 Centro Tecnológico de la Carne de Galicia, Rúa Galicia No. 4, Parque Tecnológico de Galicia, San Cibrao das Viñas, 32900 Ourense, Spain; mirianpateiro@ceteca.net (M.P.); jmlorenzo@ceteca.net (J.M.L.); 3 Área de Tecnología de los Alimentos, Facultad de Ciencias de Ourense, Universidad de Vigo, 32004 Ourense, Spain

**Keywords:** extraction, pectin, pasting, response surface methodology, starch, sugar beet pulp, viscosity

## Abstract

The objective of this study was to characterize the properties of pectin extracted from sugar beet pulp using subcritical water (SWE) as compared to conventional extraction (CE). The research involved advanced modeling using response surface methodology and optimization of operational parameters. The optimal conditions for maximum yield of pectin for SWE and CE methods were determined by the central composite design. The optimum conditions of CE were the temperature of 90 °C, time of 240 min, pH of 1, and pectin recovery yield of 20.8%. The optimal SWE conditions were liquid-to-solid (L/S) ratio of 30% (*v/w*) at temperature of 130 °C for 20 min, which resulted in a comparable yield of 20.7%. The effect of obtained pectins on viscoamylograph pasting and DSC thermal parameters of corn starch was evaluated. The contents of galacturonic acid, degree of methylation, acetylation, and ferulic acid content were higher in the pectin extracted by SWE, while the molecular weight was lower. Similar chemical groups were characterized by FTIR in both SWE and CE pectins. Color attributes of both pectins were similar. Solutions of pectins at lower concentrations displayed nearly Newtonian behavior. The addition of both pectins to corn starch decreased pasting and DSC gelatinization parameters, but increased ΔH. The results offered a promising scalable approach to convert the beet waste to pectin as a value-added product using SWE with improved pectin properties.

## 1. Introduction

Sugar beet (*Beta vulgaris* L.) pulp (SBP), a by-product of the sucrose producing factory, undergoes processing steps including pressing and drying. Pulp drying is an energy-consuming process and uses about one-third of the whole energy needs of a sucrose factory [1]. SBP is a valuable by-product, but it is commonly used as animal feed or dumped in landfill in regions with no livestock farming [2]. Researches have been previously accomplished to use this renewable byproduct as pectin precursors [3,4]. Sugar beet pulp pectin (SBPP) is a biopolymer that consists of polygalacturonic chains, methyl, and acetyl ester groups as well as neutral sugars and phenolic compounds expressed as ferulic acid [5,6]. Pectin is conventionally obtained from vegetable materials using a hot dilute mineral acidic solution [7]. Conventional acid extraction of pectin is a time-consuming process using harsher extraction conditions of temperature and pH, which cause the degradation of pectin and de-esterification of the polygalacturonic chain [6]. Furthermore, such procedures may result in environmental pollution by producing acidic wastewater [8]. 

The shortcomings of these methods have stimulated interest in alternative processing technologies, which preserve the quality of the extracted compounds [9,10,11]. Subcritical water extraction (SWE) is a simple and rapid hydrolysis method as compared to acid and enzymatic hydrolysis, which is considered as a non-conventional extraction method (eco-friendly technology) that uses water in a sub-critical state (100–374 °C at 0.10–22 MPa) as solvent [12]. This technology facilitates the extraction process without any need for pre-treatment, shortens reaction time, and reduces residues generation [13,14,15]. Only a few attempts have been made to utilize SBP for the production of pectin using subcritical water extraction (SWE) [16]. Chen e al. [16] used combined methods of SWE and ultrasonic assisted-extraction (UAE) to obtain pectin-enriched material (PEM). They optimized the extraction parameters to obtain the highest extraction yield of 24.6%. PEM solution showed a non-Newtonian behavior, and the addition of PEMs to native maize starch significantly decreased the pasting parameters and increased the onset gelatinization temperature and enthalpy. In their study, both SWE and UAE methods were used in combination, making it difficult to understand which pretreatment is responsible for the observed results. Thus, it is important to optimize the extraction process, to understand the interactions between the different parameters during extraction, and to maximize the pectin yield. The response surface methodology (RSM) is a tool that allows through mathematical and statistical models to optimize processes with multiple variables. In addition to taking into account the interaction between the variables, it allows to decrease the number of experimental trials, development time, and total cost [17,18].

Our study aimed at using SWE as pretreatment for obtaining pectin from SBP. Thus, the main objective of this study was to determine and optimize SWE operating conditions to recover highest yield of pectin from SBP using RSM. In addition, chemical, physical, and rheological properties of the pectin obtained under the optimal condition were examined, comparing them with those obtained by conventional extraction.

## 2. Results and Discussion

### 2.1. Chemical Composition of SBP

The results of the composition analysis of SBP showed that low ash content (0.3% *w/w*) was present in SBP. This result suggests there was not much inorganic matter in the pulp. The protein content of SBP was 6.1% DW (dry weight), which was in agreement with the values (6–10.3%) found in the literature [6,19]. The amount of lipids in SBP was 0.2% DW. The amount of crude fiber was 73.37% DW, a value close to those determined in the pulp by Olmos and Hansen [19].

### 2.2. Modeling and Optimization of Operational Parameters

The pectin yield depends on pH, temperature, and time extraction conditions [20]. RSM was used for extraction yield modeling and determination of the optimal conditions for SWE and CE pectins. Optimization of the extraction variables was carried out using CCD. The obtained range of SWE yield at different times, temperatures, and liquid-to-solid (L/S) ratios, which were applied in the CCD, was 11.75–20.55%. The R^2^, adj R^2^, and CV values were 0.98, 0.96, and 2.44, respectively, indicating the applicability of the model. Properties of the selected model for SWE and CE methods were presented in Table 1.

In the case of SWE, the value of R^2^ (0.98) implied that this model could explain a high percent of the variation in the observed data. According to CCD, the optimal conditions of SWE were at levels 1, −1, and −1 (codded), showing that a temperature of 130 °C, extraction time of 20 min, and L/S ratio of 30% (*v/w*) were the optimal conditions for SWE method. The verification of the experiments was conducted under optimal conditions in order to compare the predicted and the actual values of yield. The actual values (*n* = 3) were yield = 20.65, with 0.97 for the value of the desirability function. The highest yield, which achieved on these optimized conditions, was 20.65%. The rather low pectin yield could be attributed to the applied pressure and temperature of the extractor used for SWE in our study. Chen et al. [16] reported extraction yield of pectin using ultrasonic-assisted extraction (UAE) combined with SWE using similar extraction conditions (L/S of 44.03%, 120.72 °C for 30.49 min) in the range of 17.3–24.87%, which differ a little from our results. The applied high pressures (10 MPa) besides ultrasound pretreatment used in combination with SWE yielded higher pectin in their study, whereas we obtained similar maximum pectin yield by using only SWE method.

The analysis of variance (ANOVA) results of the suggested model for the conventional extraction based on CCD (Table 1) noticed that the obtained regression model was significant (*p*-value = 0.0003), while lack-of-fit value was insignificant (*p*-value = 0.6), showing that the suggested model was significantly well-fitted. Additionally, an important correlation degree was observed between experimental and predicted data with an R^2^ value of 0.91. In the case of SWE, analysis of variance showed that the generated model was significant (*p* < 0.0001) and the lack-of-fit was insignificant (*p* = 0.1). The insignificant lack-of-fit is a good sign for model fit for the SWE method.

**Figure 1:**
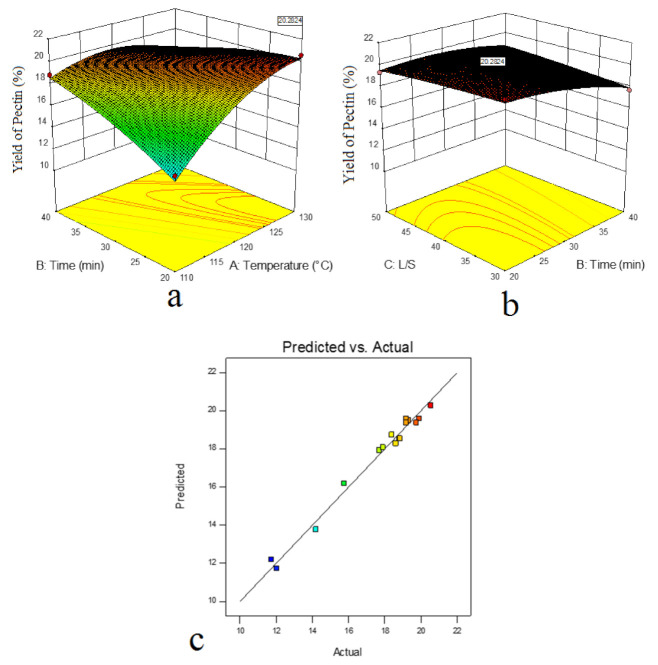
Response surface plots showing the effect of the interaction: (**a**) between temperature and time (L/S ratio: 0), (**b**) between time and L/S ratio (temperature: 0), (**c**) correlation between predicted and actual values.

Figure 1 demonstrates response surface plots showing the effect of the interaction (a) between temperature and time (L/S ratio = 0) and (b) between time and L/S ratio (temperature = 0). As shown in this figure, the pectin yield increased significantly when the extraction temperature increased. It could be ascribed to the lower dielectric constant of water, which favors dissolving pectin in water [21]. The ellipse-shaped region in the contour plot was indicative of the interaction effect between temperature and time (Figure 1a). The maximum yield of pectin was achieved in the center of the ellipse shape, which was equivalent to higher temperatures and longer times, and decreased from center to the boundary, which was equivalent to lower temperatures and shorter times.

The steepness of response surface according to extraction time (B) and L/S ratio (C) showed that extraction time and L/S ratio had a great influence with the change of extraction conditions (Figure 1b). Furthermore, the ellipse shape in the contour plot (Figure 1b) indicated the interaction effect between time and L/S ratio. The maximum yield of pectin was achieved in the center of the ellipse shape (longer times and lower L/S ratios) and decreased from center to boundary (shorter times and higher L/S ratios). The reason for the reduction of pectin yield with increasing the L/S ratio was that more solvent raised the solving volume; however, too much solvent led to the additional load for further treatment [22]. Figure 1c depicted the correlation between actual and predicted yield values. Both temperature and time showed positive quadratic effects (*p* < 0.05), while the L/S ratio showed a negative linear impress on the pectin yield (*p* < 0.05).

For the CE pectin yield, analysis of variance of the model showed that the generated model was significant (*p* = 0.0003) and the lack of fit was insignificant (*p* = 0.66). The R^2^, adj R^2^, and CV values were 0.91, 0.84, and 6.76, respectively, indicating that the model can be used to navigate the design space. Optimization of conventional extraction conditions indicated that time and temperature showed positive, while the pH showed a negative, linear impact on the pectin yield. Extraction temperature (70–90 °C), extraction time (2–4 h), and pH (1–1.5) were considered as the conventional extraction variables. The ratio of solid to liquid was chosen to be constant (1:20) for all treatments [3]. The optimum conditions of conventional extraction were estimated to be a temperature of 90 °C, an extraction time of 4 h and a pH of 1. Extraction using these optimized conditions provided the highest yield of 20.38%. This recovery yield was higher than that found by Huang et al. (2018), who observed a yield around 17.5% using similar extraction conditions (pH 1.5, 90 °C for 2 h). The verification of experiments was conducted under optimal conditions. The actual values (*n* = 3) were yield = 20.38, with 0.93 for the value of the desirability function. Extraction using the optimized conditions of conventional process achieved the best yield of 20.38%. The optimal conditions of conventional acid extraction include the highest temperature and time, and lower pH values.

The obtained range of conventional extraction yield at times, temperatures, and pH values, which were applied in the CCD, were 11.9–22.46%. It could be attributed to the pH of the suspension and the temperature of the water bath used for conventional extraction. Time (*p* > 0.05) and temperature (*p* < 0.05) showed positive linear impacts on the yield of pectin, while the pH showed a negative linear impact on the yield (*p* < 0.05). The extraction temperature exerts a linear effect on the pectin yield. Hence, the yield increased linearly with the increase in the extraction temperature within a range of 60–100 °C. To sum up, SWE was more efficient for pectin extraction than CE method, reporting the same yields (SWE = 20.65% vs. CE = 20.38%) in a much shorter time (SWE = 20 min vs. CE = 240 min). In other words, the yield of SWE pectin for the extraction time of 20 min gained 20.65%, which is not significantly different from the CE method’s yield (20.38%) after 240 min. Thus, by the application of subcritical water only for 20 min, the pectin extraction accelerated in comparison with the conventional extraction.

### 2.3. Characterization of Pectin

#### 2.3.1. FTIR Characterization

**Figure 2:**
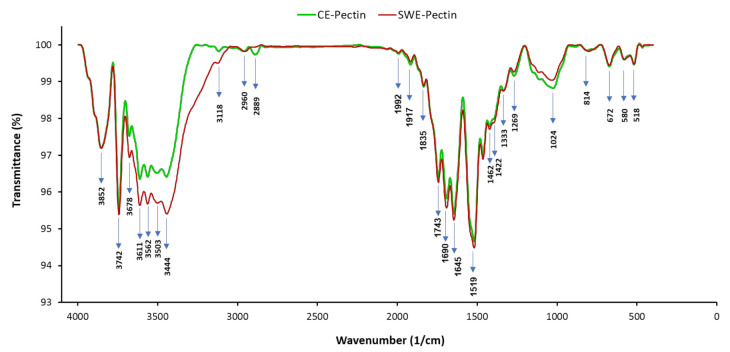
FT-IR spectra of conventionally extracted pectin and SWE pectin.

The infrared spectroscopy is a fast and convenient method for investigation of functional groups of polysaccharides. In order to confirm the pectin identity and the effect of SW on the structural properties of extracted pectin, the sample was analyzed by Fourier Transform Infrared Spectroscopy (FT-IR) and compared to the spectra of conventionally extracted pectin. FTIR allowed the determination of similar chemical groups in the SW and conventional acid extracted pectin. Figure 2 represented the transmittance spectra of the pectins obtained by conventional acid extraction and SWE. Based on Manrique and Lajolo [23], bands present between 1760–1745 cm^−1^ indicated the ester carbonyl groups (COOR), and bands between 1620 and 1640 cm^−1^ indicated carboxylic ion (COO^−^). Both conventional acid extraction and SWE pectins spectra exhibited bands at around 1518 and 1743 cm^−1^, which refer to the COOR and COO^−^ groups. A broad absorption band found at 3400–3800 cm^−1^ on both of the extracted pectins was related to the stretching vibration of O–H bonds. As shown in Figure 2, the infrared spectra of SWE pectin were almost the same as conventionally extracted pectin due to their similar chemical composition.

#### 2.3.2. Chemical Analysis

The chemical composition of pectin obtained under the optimal conditions is shown in Table 2. The higher galacturonic acid (GalA) content (73%, *w/w*) indicates the more pure pectin recovered from SWE compared to CE procedure, suggesting the great ability of this extraction technology to release pectin [24]. GalA contents of SWE (73% *w/w*) and CE (68% *w/w*) pectins were both higher than the value reported by Huang et al. [20] for pectin extracted from SBP by traditional heating method (45% *w/w*). Comparing these results with those obtained with other emerging extraction techniques, similar contents (73–76% *w/w*) were found by other authors in pectin obtained from fig (*Ficus carica* L.) skin by ultrasound-microwave assisted extraction [17]. In contrast, the combination of this environmentally friendly method with other emerging technologies (ultrasonic-assisted extraction, UAE technology) failed to improve the extraction efficiency (59%) [16]. Based on the degree of methylation (DM) results shown in Table 2, a DM value more than 50% (mole) was obtained for both pectins, indicating that both CE and SWE pectins were high methoxy pectins (HMP). The degree of acetylation (DA) for CE and SWE pectins was 19.6% (mole) and 26.0% (mol), respectively. The higher DM and DA values for SWE pectin compared to those for CE pectin may suggest the good rheological properties and antioxidant activity of this pectin, and its great potential application in the food industry [21]. The ferulic acid (FA) content of CE and SWE pectins was 0.8 and 1.9 (% *w/w*), respectively. Pectins with high feruloyl groups tend to form stronger cross links [25]. As shown in Table 2, the molecular weight (MW) of SWE pectin (23.51 kDa) was significantly lower than that of CE pectin (102.27 kDa), which is possibly due to the hydrolysis and decomposition of pectin under subcritical temperature and pressure conditions with a dielectric constant, thus leading to a smaller Mw in SWE method [26]. The results were similar to the degradation behavior of cellulose and hemicellulose in subcritical water treatment [27]. The range of MW for pectin depends on its source and extraction procedure, and can significantly affect its emulsifying properties [28]. In this way, SWE methods displayed lower MW values than those obtained by other extraction methods [29,30].

### 2.4. Functional Properties of Pectin

#### 2.4.1. Color Attributes

The pectin color is a critical quality parameter for applications in food industry, preferring light-colored or colorless pectins for commercial applications [17].

The lightness (L*), redness (a*), and yellowness (*b) values of SWE and conventionally extracted pectin were shown in Table 2. The values of the color parameters of SW extracted pectin are close to the color parameters of pectin extracted from the same raw material by conventional acid hydrolysis, displaying a darker appearance (44.9 vs. 46.0) and b* (29.4 vs. 21.1) values. The obtained L* values were similar than those found by Idrovao-Encalada et al. [31] in pectins from carrot leftovers.

Pectins from SBP are generally characterized by dark-color and have a high content of phenolic material [32]. The polyphenols trapped within the pectin matrix during precipitation could give place to an increase in a* and b* values and a decrease in L* values [17].

#### 2.4.2. Rheological Measurements

**Figure 3:**
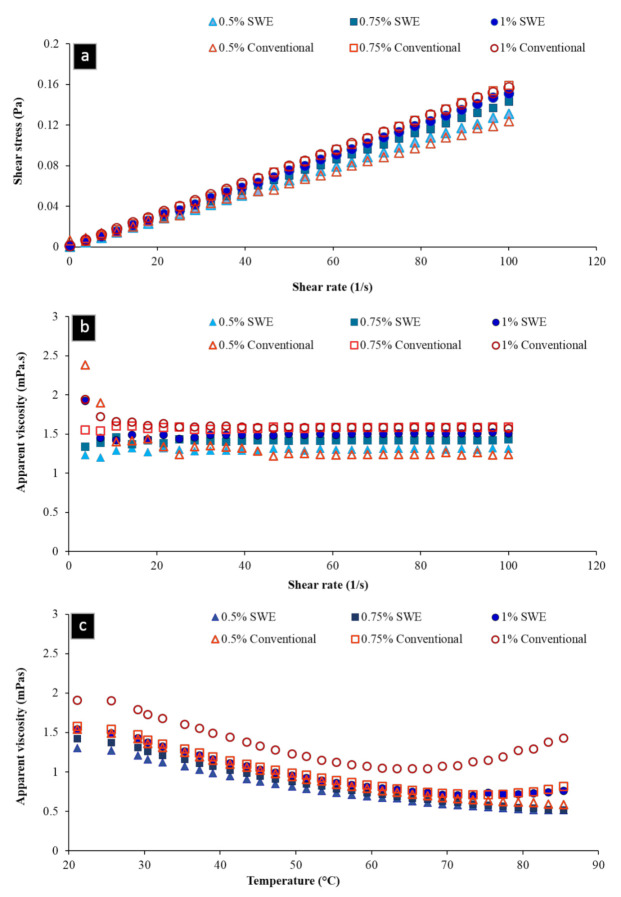
Shear stress of pectin solutions as a function of shear rate (**a**), shear-rate dependence of viscosity for various pectin solutions, (**b**) and temperature dependence of viscosity for various pectin solutions (**c**).

The pectin sample obtained under optimal extraction condition was used for rheological tests. Shear stress and apparent viscosity of pectin extracted by SWE and conventional heating as a function of shear rate at concentrations of 0.5%, 0.75%, and 1%, *w/w* were shown in Figure 3. Figure 3a demonstrates the flow behavior of pectin solutions under steady shear conditions, which were characterized by a plot of stress versus shear rate. Almost a linear trend of shear stress as function of shear rate was seen for both SWE and conventionally extracted pectins, indicating nearly Newtonian behavior, since very dilute pectin solutions (of very low viscosity) appear to have the properties of Newtonian fluids. For both pectin types, a 1% solution exhibited higher values of stress than those of lower concentration solutions. The effect of solution concentration on apparent viscosity at different shear rates is shown in Figure 3b. Higher apparent viscosity values (Figure 3b) were observed for both pectin types at higher concentration (1% *w/w*). In all solutions, viscosity was decreased with increasing the shear rate, showing a shear-thinning behavior. These observations were in agreement with the results found for pectin extracted from sugar beet pulp in other studies [16,33], and it is characteristic of random coil polysaccharides [34].

As can be observed in Figure 3b, the range of apparent viscosity between infinite shear rate viscosity (η∞) and zero shear rate viscosity (η_0_) increased from 1.2 to 1.52 mPa·s for SWE pectin solutions, and increased from 1.22 to 2 mPa·s for conventionally extracted pectin solutions with an increase in concentration from 0.5% to 1% *w/w*. One explanation for this trend could be that higher pectin concentration caused a nearly shear thinning behavior [16]. The higher viscosity values for conventionally extracted pectin solutions may be due to the protein or other hydrocolloids that remained in the water-soluble fraction during purification of pectic substances.

The temperature dependence of the apparent viscosity of pectin solutions was also investigated. The apparent viscosity of both pectin types at a constant shear rate of 50 s^−1^ is shown in Figure 3c. There is a nonlinear decrease in viscosity, especially at higher concentrations of pectin solutions, when temperature increased from 25 to 85 °C. These results are consistent with the those observed by Min et al. [34], which could be attributed to the lower molecular interactions at higher temperatures. There is an increase in viscosity of 1% and 0.75% CE pectin solutions after exceeding temperature about 70 °C. The possible explanation for this is probably due to the higher viscosity of solutions resulting from higher concentrations.

#### 2.4.3. Pasting Properties

The pasting properties of the mixtures containing corn starch with water or corn starch/pectin with water at a concentration of 1% (Table 3) showed that all the pasting parameters of corn starch (except for hot paste viscosity) decreased with the addition of pectin. Addition of pectin caused a decrease in the pasting temperature (PT), which implies faster swelling. The lower pasting temperature was ascribed to the possible interaction between starch granules and added pectin, especially at higher pectin concentrations (1%). Hydrocolloids could promote starch granules swelling capacity, likely due to the inhibition of starch components such as amylose from leaching out the starch granules into the continuous phase of paste during gelatinization [35]. In our study, pectin resulted in decrease in PT, PV, BD, FV, and SB viscosity parameters, which is disagreement with reported results by Gularte and Rosell [36] and Dartois et al. [35], as they used lower concentrations of hydrocolloids (i.e., 0.2%.). This can be explained by higher concentration (1%) of pectin used in our study; as also explained in other studies [37,38], higher concentrations of hydrocolloids are responsible of the reverse effect of hydrocolloids incorporation on pasting properties, since the starch–hydrocolloid network stability is highly dependent on the hydrocolloid concentration. This could explain reduced pasting properties of starch and pectin pastes.

Another elucidation for this result was that increasing the temperature caused hydration and blistering of the corn starch granules; thus, the concentration of the pectin in the fluid phase increased, and subsequently, the viscosity of the fluid phase increased. The relatively low peak viscosity (PV), due to the addition of pectin, implied that the mixture was suited for products requiring low gel strength and elasticity. The viscosity at the beginning of cooling or hot paste viscosity (HPV) of the corn starch pectin mixture increased in comparison to the corn starch. Final viscosity (FV) involves the molecular element present in the phenomenon of retrogradation with the beginning of molecular re-association together with increasing the viscosity. Reduction in FV indicated that the product contained corn starch, and added pectin could present viscous quality after cooking or cooling and also resist to shear stress during stirring [39]. Accordingly, the corn starch with added pectin had greater resistance to retrograde as compared to the corn starch (Table 3). The lower breakdown (BD) and setback (SB) values of the starch–pectin mixture, compared to corn starch–water was due to the prevention of granule swelling and keeping granule integrity. The lower SB value indicated that the corn starch pectin mixture would hardly be retrograded over keeping in the refrigerator.

#### 2.4.4. Thermal Properties

The effects of SWE pectin at a concentration of 1% on the thermal properties of corn starch were investigated and results were illustrated in Table 3. There were significant differences (*p* < 0.05) between onset temperature (T_O_), gelatinization peak temperature (T_P_), and complete temperature (T_C_) of corn starch with or without the addition of pectin. Therefore, the presence of pectin influenced the gelatinization characteristics of starch. A significant increase in T_O_ was observed with the addition of CE pectin solution, which is in agreement with the results found by Chen et al. [16]; however, there was no significant effect of SWE pectin on onset temperature (T_O_). The T_P_ and T_C_ of the corn starch and SWE pectin mixture were lower than those of corn starch (Table 4). The phase transition temperature range (T_C_ − T_O_) also decreased. The possible explanation for this trend was that the association of SWE pectin and starch immobilize water molecules and reduce free water to starch ratios [40,41], and as a consequence, the mass and heat transfer rates decline [42]. The enthalpy change (ΔH) increased with the addition of pectin, which differs from the results found by other authors [16]. Higher enthalpy of gelatinization meant more energy required for complete removal of water. Therefore, the high peak temperatures observed in the starch samples containing the conventionally extracted pectin as well as the higher gelatinization enthalpy of these samples showed that these samples had a better ability to retain water.

## 3. Materials and Methods

### 3.1. Materials

The wet sugar beet pulp (SBP) came from the pulps transported to the pulp press in the Khoy sugar factory (Khoy, East Azerbaijan Province, Iran). All sugar beets used in this factory were of Flores and Virginia varieties. Starch from corn (practical grade, S4180 Sigma-Aldrich, Munich, Germany) was used for pasting and thermal analysis.

### 3.2. Chemical Analysis of SBP

The moisture, protein, and ash contents of SBP were determined using AACC methods of 44.15 A, 46.13, and 08.01, respectively. Lipid and crude fiber contents were also determined according to the AACC methods of 30.10 and 32.10, respectively [43].

### 3.3. Preparation and Extraction

#### 3.3.1. Preparation of SBP

SBP was oven-dried at 40 °C (moisture content less than 3%), ground using a desktop hammer mill (Glencrestone Ltd., Castleford, UK) and sifted through 60 mesh sieve.

#### 3.3.2. Subcritical Water Extraction of Pectin

**Figure 4:**
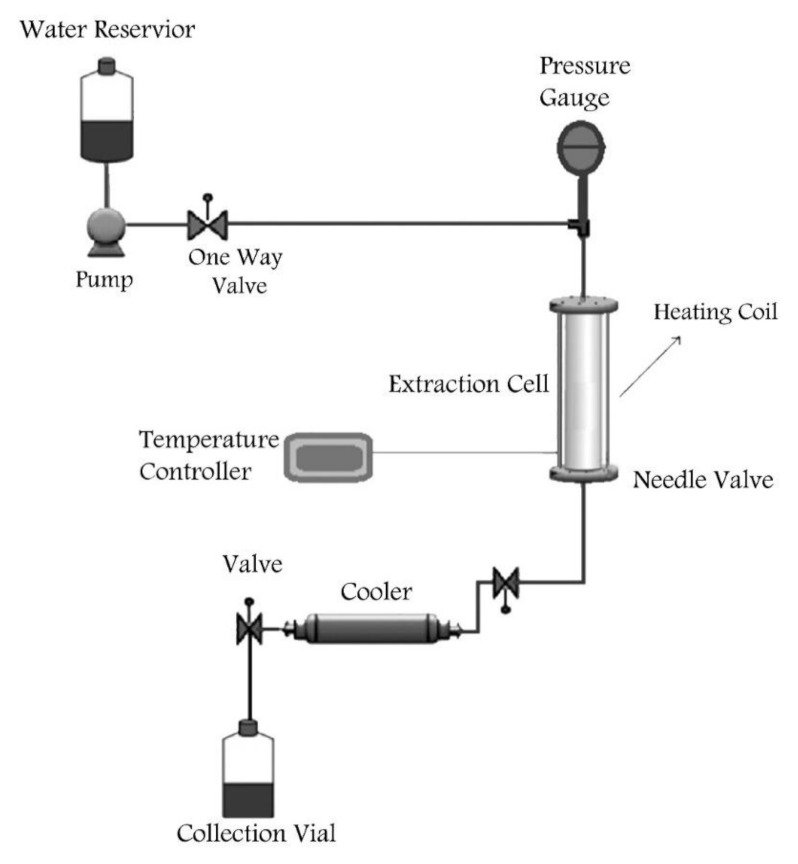
Schematic presentation of laboratory-built subcritical water extraction system.

The extraction of pectin from the dried pulp (moisture content less than 3%) was carried out using a laboratory-built batch-type instrument (Figure 4).

The different parts of this system were a deionized water feed tank, a high-pressure metering pump (Comet type: MTP AX 2/70 m), an extraction vessel (70 mL) with heating wires wrapped around it, and a thermometer. The maximum pressure and temperature of the system were 5 MPa and 220 °C, respectively. The extraction vessel was heated before each run and the temperature was set to the desired value for extraction. When the temperature reached to pre-set value, extraction time was counted. After extraction, the obtained extract was filtered through a filter paper (20 μm) and chilled in an ice receptacle, then was precipitated with 2 volumes of anhydrous ethanol for 1 h. After that, a centrifugation at 15,000 rpm for 20 min was undertaken, and the assembled pectin was vacuum dried in an oven at 40 °C for 24 h. 

The extraction yield was measured using the equation below:

(1) $$\mathrm{Yield}\;\left(\%\right)=\;\frac{\;\mathrm{Dried}\;\mathrm{pectin}\;\mathrm{material}\;\mathrm{weight}}{\;\mathrm{Dried}\;\mathrm{SBP}\;\mathrm{powder}\;\mathrm{weight}}\;\times 100$$

#### 3.3.3. Conventional Extraction of Pectin

The conventional extraction (CE) of pectin from the dried pulp (moisture content less than 3%) was carried out according to the method described previously [44]. 

#### 3.3.4. Experimental Design

Response surface methodology (RSM) was used to determine the integrated effects of three independent variables, extraction temperature (X_1_), extraction time (min) (X_2_), and liquid-to-solid (L/S) ratio (X_3_), on the yield of pectin recovered by SWE, and extraction temperature (X_1_), extraction time (h) (X_2_), and pH (X3) were considered as CE variables. Thus, a three-variable-three-level central composite design (CCD) consisting of 20 experimental runs was used to determine the optimal conditions for maximum yield of pectin achieved (Table 4). This design included six replicates (trials 7, 9, 11, 13, 16, 17) at the center point. The fitness of the polynomial model equation was evaluated using lack of fit, coefficient of regression (R^2^), adequate precision, and standard deviation. The effect of variables was displayed in 3D response surfaces.

RSM comprises a group of empirical techniques devoted to the evaluation of relations existing between a series of controlled experimental factors and measured responses, according to one or more selected criteria [45]. This technique is a widely used mathematical and statistical method for modeling and analyzing a process in which the response of interest is affected by various variables and the objective of this method is to optimize the response. The parameters that affect the process are called independent variables, while the responses are called dependent variables [46]. The advantages offered by the RSM can be summarized as determining the interaction between the independent variables, modeling the system mathematically, and saving time and cost by reducing the number of trials [47]. RSM was used for determining optimum condition and the integrated effects of three independent variables: a central composite design (CCD) inclusive of 20 experiential trials with six repeats at the center point was used for SWE and conventional extraction.

#### 3.3.5. Purification of Pectic Substances

In order to eliminate the water-insoluble fraction (WIF), which could consist of cellulose, hemicellulose, and lignin, the pectic substances extracted from the conventional procedure or subcritical water at the optimum conditions were purified by a centrifugation process. The dried pectic substances were dissolved in fresh Milli-Q water (1:200, *w/v*), prepared from a Milli-Q water purification system (Millipore Co., Milford, MA, USA), for 15 h and centrifuged at 9072 g during 20 min at room temperature in a LABOFUGE ll centrifuge (Ollmann and Co. KG, LABSCO, Friedberg, Germany) to remove WIF. The supernatants were filtered through 11 and 3 µm Millipore membranes (Millipore Co., Milford, MA, USA), respectively. The filtrates were oven-dried at 40 °C for 12 h to recover the “purified” pectin, and weighed. The obtained pectins were used for the characterization.

### 3.4. Physico-Chemical Analysis of Pectin

#### 3.4.1. FTIR Characterization of Pectin

The pectin identity and the effect of SW on the structural properties of extracted pectin were determined through infrared spectra of pectin with a KBr (alkali halogenide)-pellet technique using a Tensor 27 FTIR spectrometer (BrukerOptik, Gmbh, Berlin, Germany) at the range of 400–4000 cm^−1^. Wave number accuracy was 0.1 cm^−1^. All spectra were post-processed using OPUS software (Version 6.5, Bruker Optik, Berlin, Germany).

#### 3.4.2. Determination of Galacturonic Acid

The galacturonic acid (GalA) content of pectin was determined using high-performance anion exchange chromatography with a pulsed amperometric detector (HPAEC-PAD) according to the method described by Garleb et al. [48], which was earlier reported [44]. The GalA content was expressed as weight fraction of total pectin weight (*w/w*%).

#### 3.4.3. Degree of Methylation (DM) and Degree of Acetylation (DA)

Thirty milligrams of pectin sample was suspended in 1 mL of isopropanol-water mixture (1:1, *v/v*), containing 0.4 M sodium hydroxide, and held at a temperature of 4 °C for 2 h. The suspension was then centrifuged, and 20 μL of the clear supernatant was injected in the column [49,50]. The saponification led to the release of esterified methoxy and acetyl groups, which then were quantified injecting 20 μL in a WellChrom system HPLC equipped with a RI detector and a Nucleodur C18 Pyramid column (4.6 × 250 mm, 5 μm particle size). The mobile phase consisted in HPLC-grade water adjusted to pH 3.5 with sulfuric acid. The chromatography conditions were as follows: flow rate of 0.7 mL/min at 25 °C. Degrees of methoxylation (DM) and acetylation (DA) were expressed as the percent molar ratio of methanol (MeOH) or acetic acid (AcOH) to GalA, respectively.

#### 3.4.4. Determination of Ferulic Acid Content

The ferulic acid content was determined by HPLC after saponification and extraction according to Yapo et al. [6]. Pectin sample (50 mg) was saponified with 3 mL of NaOH 2 M under nitrogen stream in dark at room temperature conditions, overnight. Then, it was neutralized to pH 2 with HCl 2M to continue with phenolic compounds extraction in ethyl acetate (3 × 3 mL). The ethyl acetate phase was evaporated under nitrogen at room temperature and the residue dissolved in 1 mL of methanol/H_2_O (1:1, *v/v*). Twenty-five microliters of this material were injected into a WellChrom system HPLC equipped with a Perfectsil Target ODS-3 C18 column (4.6 × 250 mm, 5 μm particle size) coupled on line with an UV detector. Elution was carried out with 75% solvent A (Milli-Q water + 0.05% TFA) and 25% solvent B (acetonitrile + 0.05% TFA) at constant temperature of 25 °C and at a flow rate of 1 mL/min. Ferulic acid (purity > 99%, Sigma–Aldrich Corp., St. Louis, MO, USA) was used as an external standard. The ferulic acid content was expressed as weight fraction of total pectin weight (*w/w*%).

#### 3.4.5. Molecular Weight (MW)

The molecular weight was determined by gel permeation chromatography (GPC), in combination with a high-performance liquid chromatography instrument (Shimadzu LC-20A, Kyoto, Japan) equipped with an Ultrahydragel Linear column (7.8 × 300 mm, 10 μm particle size). The purified pectin solution was centrifuged and passed through 0.45 μm and 3 μm filters; 50 μL of this solution were applied to a gel-filtration chromatographic column maintained at a temperature of 35 °C, eluted with NaNO_3_ 0.1 M in water at a flow rate of 1 mL/min and detected by a refractive index detector. Six polysaccharides of MW of 12,200 to 830,000 g/mole including T1.5, GPC Standard 50, Glucose, T40, T10, and T750 were used for the calibration of the column.

### 3.5. Functional Properties of Pectin

#### 3.5.1. Color Measurement

Pectin color was measured in the CIELAB space using a Minolta CR-300 portable colorimeter (standard illuminant D65) in terms of L* value (lightness), a* value (redness and greenness), and b* value (yellowness and blueness), as an average of three measurements at three different locations. The instrument was standardized against a white RAL color (1013) (L* = 87.15; a* = 0.22, and b* = 10.5) before measurements. From these values, the total color difference (ΔE) was calculated according to the following equation:

(2) $$\Delta E=\sqrt{{\left(L^{*}-L_{0}^{*}\right)}^{2}+{\left(a^{*}-a_{0}^{*}\right)}^{2}+{\left(b^{*}-b_{0}^{*}\right)}^{2}}$$

where L_0_, a_0_, and b_0_ are color values of RAL color (1013). The comparison was carried out between lab color values of conventionally and SW extracted pectin.

#### 3.5.2. Rheological Measurements

In order to define rheological characteristics, three different concentrations (0.5%, 0.75%, and 1%, *w/w*) of the two pectins obtained under conventional or SW extraction, were loaded onto a strain-controlled rheometer Anton Paar (DG26.7) with a 40 mm concentric cylindrical geometry, which was joint with a temperature-controlled water bath. Steady shear measurements were made in a range of shear rates from 0.1 to 100 s^−1^ at 25 °C.

#### 3.5.3. Pasting Property Measurement and Thermal Analysis

The influence of adding pectin on pasting characteristics of normal corn starch was measured according to the method described by Chen et al. [16] with slight modifications. The moisture content of the normal corn starch was determined according to AACC A15-44 method. Then, the correction of moisture content based on 14% was applied and the corn starch amount needed to perform the test was weighted. Sample was prepared by mixing corn starch and 100 mL of distilled water/pectin solutions (1% *w/w*). The mixture was stirred manually for 1 min. The pectin-induced changes in pasting properties of normal corn starch were determined with a Brabender viscoamylograph (4.1.0) with a 700 cmg cartridge fitted with the rotation speed set at 75 RPM. The heating and cooling cycles were programmed in the following manner: the sample was heated at 7.5 °C/min from 30 to 95 °C, then held for 20 min at 95 °C, cooled to 50 °C at 7.5 °C/min, and then held for 20 min at 50 °C. The influence of adding pectin on thermal characteristics of normal corn starch was measured according to the method described by Chen et al. (2015) with slight modification. To identify how extracted pectins change the thermal characteristics of normal corn starch, differential scanning calorimetry (NETZSCH DSC200F3) was used. Normal corn starch (4 mg) was weighed into stainless steel pans, distilled water (20 μL) or pectin solution (1% *w/w*) was added, and the pans were hermetically sealed. After 1 h at 25 °C, the sample was heated from 30 to 95 °C at 5 °C/min. The DSC instrument was calibrated with indium and distilled water.

Normal corn starch (Sigma Aldrich, S4180) was used for measuring pasting properties (pasting temperature (Tp), peak viscosity (PV), hot paste viscosity (HPV), final viscosity (FV), breakdown (BD) and setback (SB)) and thermophysical characteristics of starch with and without conventional or SW extracted pectin.

### 3.6. Statistical Analysis

All experiments were done in triplicates. Optimization was carried out using Design-Expert software, version 10 (Stat-Ease, Inc., Minneapolis, MN, USA). Normal distribution and homogeneity of variance were previously tested (Shapiro–Wilk). The results of thermodynamic properties were assessed with the Independent-Samples T-Test, using the statistical software SPSS 16.0 (SPSS Inc., Chicago, SPSS Inc., Chicago, IL, USA).

## 4. Conclusions

The purpose of this research was to use the subcritical water fluid as a novel technology and ecofriendly way for the extraction of pectin from SBP to maximize pectin extraction efficiency. The optimum extraction conditions by SW were as follows: temperature of 130 °C, time of 20 min, and liquid-to-solid (L/S) ratio of 30 (*v/w*). The maximum yield of pectin achieved in optimum conditions was 20.65%. The optimum conditions of conventional extraction were a temperature of 90 °C, time of 4 h, and pH of 1, and the highest pectin yield using these optimized conditions was 20.38%. SWE was more efficient than CE method, reporting the same extraction yields (SWE = 20.65% vs. CE = 20.38%) in a much shorter time (SWE = 20 min vs. CE = 240 min). Therefore, SWE was nearly 12 times faster than CE method. The rheological measurements indicated that the pectin solution exhibited Newtonian behavior in the concentration range of 0.5–1%. Addition of pectin decreased pasting parameters (except hot paste viscosity) and DSC gelatinization parameters of starch (T_P_, T_C_, and T_C_ − T_O_), while shifted ΔH to higher values. The T_O_ of starch was minimally affected by the addition of pectin. Brabender viscoamylograph results were in good agreement with the DSC results. The results offered a promising approach to convert the beet waste to a value-added product such as pectin. SWE as a green procedure can reduce pectin extraction time on an industrial scale. In addition, this method facilitates the achievement of the pectin with improved specifications.

## Figures and Tables

**Table 1:** Estimated regression coefficients for the model and the analysis of variance (ANOVA) for the experimental data of conventionally extracted pectin yield.

Conventional Extraction			SWE		
Term	Coefficient ^1^	Yield	Term	Coefficient ^1^	Yield
Intercept	β0	16.80	Intercept	β0	19.37
X1	β1	1.08 **	X1	β1	1.79 ***
X2	β2	0.62	X2	β2	1.00 ***
X3	β3	−2.74 ***	X3	β3	−0.31 *
X1^2^	β11	−0.11	X1^2^	β11	−1.43 ***
X2^2^	Β22	−0.54	X2^2^	Β22	−0.52 **
X3^2^	Β33	0.15	X3^2^	Β33	−0.10
X1 (Temperature) × X2 (Time)	β12	0.26	X1 (Temperature) × X2 (Time)	β12	−1.78 ***
X1 (Temperature) × X3 (pH)	β13	−0.058	X1 (Temperature) × X3 (L/S ratio)	β13	0.32
X2 (Time) × X3 (pH)	Β23	−0.79	X2 (Time) × X3 (L/S ratio)	Β23	0.40 *
	R^2^	0.9158		R^2^	0.98
	Adj.R^2^	0.8401		Adj.R^2^	0.96
	F	12.09		F	68.93
	Model	0.0003 ***		Model	<0.0001 ***
	Lack of fit	*p* = 0.6		Lack of fit	*p* = 0.1
	Std. Dev	1.11		Std. Dev	0.44
	CV	6.76		CV	2.44

^1^ Yn = β0 + β1X1 + β2X2 + β3X3 + β11X12 + β22X22 + β33X32 + β12X1X2 + β13X1X3 + β23X2X3; DF, Degrees of freedom; CV, coefficient of variation. * Significant (*p* < 0.05); ** significant (*p* < 0.01); *** significant (*p* < 0.001); ns not significant (*p* > 0.05).

**Table 2:** Constituents and color parameters of pectin obtained under the optimum condition by conventional extraction (CE) and SWE.

	CE Pectin	SWE Pectin
**Constituents**		
GalA (% *w/w*)	68.15 ± 1.63	73.00 ± 1.98
DM (% mole)	57.02 ± 1.52	84.19 ± 2.07
DA (% mole)	19.55 ± 1.30	25.96 ± 2.04
FA (% *w/w*)	0.81 ± 0.05	1.87 ± 0.29
MW (kDa *)	102.27 ± 0.1	23.51 ± 0.4
**Color Parameters**		
L*	46.0 ± 1.0	44.9 ± 1.0
a*	2.1 ± 1.0	2.2 ± 0.3
b*	21.1 ± 1.0	29.4 ± 0.3
ΔE	42.5	46.3

Data are mean of triplicate measurements ± SD. GalA: Galacturonic acid, DM: Degree of methylation, DA: Degree of acetylation, FA: Ferulic acid, MW: Molecular weight. * 1 kDa = 1000 g/mol.

**Table 3:** Brabender and DSC gelatinization parameters of native starch and pectin starch mixture.

	Brabender Viscosity Parameters *						DSC Gelatinization Parameters **				
	PT (°C)	PV (BU)	HPV (BU)	BD (BU)	FV(BU)	SB (BU)	T_O_ (°C)	T_P_ (°C)	T_C_ (°C)	T_C_ − T_O_	ΔH (J/g)
Starch ***	72.4 ± 0.3 ^a^	377 ± 1.9 ^a^	210 ± 3.0 ^b^	167 ± 0.4 ^a^	483 ± 2 ^a^	273 ± 2.0 ^a^	65.7 ± 0.2 ^b^	71.3 ± 0.3 ^b^	75.8 ± 0.4 ^a^	10.1	0.80 ± 0.10 ^c^
Starch + CEP (1%) ‡	71.6 ± 0.1 ^b^	342 ± 1.0 ^c^	210 ± 0.5 ^b^	132 ± 0.3 ^b^	437 ± 1 ^b^	227 ± 2.0 ^b^	67.8 ± 0.1 ^a^	72.0 ± 0.5 ^a^	75.6 ± 0.5 ^a^	7.8	2.20 ± 0.01 ^a^
Starch + SWE Pectin 1%	70.3 ± 0.2 ^c^	355 ± 2.6 ^b^	254 ± 2.7 ^a^	101 ± 0.1 ^c^	427 ± 4 ^c^	173 ± 2.5 ^c^	65.8 ± 0.4 ^b^	70.4 ± 0.1 ^c^	74.2 ± 0.5 ^b^	8.4	1.61 ± 0.03 ^b^

‡ Starch + CEP (1%): Starch + Conventionally extracted pectin 1%; * Brabender parameters: PT: pasting temperature, PV: peak viscosity, HPV: hot paste viscosity, FV: final viscosity, SB: set back value (SB = FV − HPV), BD: breakdown value (BD = PV − HPV), BU: Brabender units. ** DSC parameters: T_O_: onset temperature; T_P_: peak temperature; T_C_: completion temperature; ΔH: enthalpy change. *** Native corn starch. The data are average of three measurements with standard deviation. Mean values in the same column with different letter are significantly different (*p* < 0.05).

**Table 4:** Experimental design of the levels, variables of central composite, and pectin yield by SWE and CE.

Run	SWE Pectin				CE Pectin			Response (Yield, %)
	Independent Variable			Response (Yield, %)	Independent Variable			
	X_1_	X_2_	X_3_		X_1_	X_2_	X_3_	
	Temperature (°C)	Time (min)	L/S Ratio (*w/w* %)		Temperature (°C)	Time (h)	pH	
1	130 (1)	20 (−1)	50 (1)	19.3	80 (0)	3 (0)	1.25 (0)	16,93
2	110 (−1)	40 (1)	30 (−1)	18.81	70 (−1)	4 (1)	1 (−1)	18.76
3	103 (−1.68)	30 (0)	40 (0)	11.75	80 (0)	3 (0)	1.25 (0)	16.19
4	120 (0)	30 (0)	57 (1.68)	18.83	90 (1)	4 (1)	1 (−1)	20.75
5	130 (1)	40 (1)	30 (−1)	17.7	80 (0)	3 (0)	1.25 (0)	15.18
6	120 (0)	30 (0)	23 (−1.68)	19.2	97 (1.68)	3 (0)	1.25 (0)	19
7	120 (0)	30 (0)	40 (0)	19.18	90 (1)	2 (−1)	1 (−1)	18.21
8	110 (−1)	20 (−1)	30 (−1)	14.2	70 (−1)	2 (−1)	1.5 (1)	13.66
9	120 (0)	30 (0)	40 (0)	19.18	80 (0)	3 (0)	1.25 (0)	18.32
10	120 (0)	13 (−1.68)	40 (0)	15.75	70 (−1)	2 (−1)	1 (−1)	17
11	120 (0)	30 (0)	40 (0)	19.75	63 (−1.68)	3 (0)	1.25 (0)	14.26
12	110 (−1)	40 (1)	50 (1)	17.9	90 (1)	4 (1)	1.5 (1)	14.48
13	120 (0)	30 (0)	40 (0)	19.75	80 (0)	1.3 (−1.68)	1.25 (0)	13.52
14	120 (0)	47 (1.68)	40 (0)	19.9	70 (−1)	4 (1)	1.5 (1)	12
15	110 (−1)	20 (−1)	50 (1)	12.04	90 (1)	2 (−1)	1.5 (1)	14.84
16	120 (0)	30 (0)	40 (0)	19.18	80 (0)	4.7 (1.68)	1.25 (0)	17.24
17	120 (0)	30 (0)	40 (0)	19.2	80 (0)	3 (0)	1.7 (1.8)	11.9
18	130 (1)	20 (−1)	30 (−1)	20.55	80 (0)	3 (0)	0.85 (−1.6)	22.46
19	137 (1.68)	30 (0)	40 (0)	18.61	80 (0)	3 (0)	1.25 (0)	16
20	130 (1)	40 (1)	50 (1)	18.4	80 (0)	3 (0)	1.25 (0)	18

## Data Availability

The data presented in this study are available in article.

## Sample Availability

Not available.

## References

[1] Asadi M. (2006). Beet-Sugar Handbook.

[2] Kühnel S., Schols H.A., Gruppen H. (2011). Aiming for the complete utilization of sugar-beet pulp: Examination of the effects of mild acid and hydrothermal pretreatment followed by enzymatic digestion. Biotechnol. Biofuels.

[3] Lv C., Wang Y., Wang L.J., Li D., Adhikari B. (2013). Optimization of production yield and functional properties of pectin extracted from sugar beet pulp. Carbohydr. Polym..

[4] Panahirad S., Dadpour M., Peighambardoust S.H., Soltanzadeh M., Gullón B., Alirezalu K., Lorenzo J.M. (2021). Applications of carboxymethyl cellulose- and pectin-based active edible coatings in preservation of fruits and vegetables: A review. Trends Food Sci. Technol..

[5] Mohnen D. (2008). Pectin structure and biosynthesis. Curr. Opin. Plant Biol..

[6] Yapo B.M., Robert C., Etienne I., Wathelet B., Paquot M. (2007). Effect of extraction conditions on the yield, purity and surface properties of sugar beet pulp pectin extracts. Food Chem..

[7] Srivastava P., Malviya R. (2011). Sources of pectin, extraction and its applications in pharmaceutical industry—An overview. Indian J. Nat. Prod. Resour..

[8] Zhang L., Ye X., Ding T., Sun X., Xu Y., Liu D. (2013). Ultrasound effects on the degradation kinetics, structure and rheological properties of apple pectin. Ultrason. Sonochem..

[9] Zhang J., Wen C., Zhang H., Duan Y., Ma H. (2020). Recent advances in the extraction of bioactive compounds with subcritical water: A review. Trends Food Sci. Technol..

[10] Ravash N., Peighambardoust S.H., Soltanzadeh M., Pateiro M., Lorenzo J.M. (2020). Impact of high-pressure treatment on casein micelles, whey proteins, fat globules and enzymes activity in dairy products: A review. Crit. Rev. Food Sci. Nutr..

[11] Soltanzadeh M., Peighambardoust S.H., Gullon P., Hesari J., Gullón B., Alirezalu K., Lorenzo J. (2020). Quality aspects and safety of pulsed electric field (PEF) processing on dairy products: A comprehensive review. Food Rev. Int..

[12] Lachos-Perez D., Martinez-Jimenez F., Rezende C.A., Tompsett G., Timko M., Forster-Carneiro T. (2016). Subcritical water hydrolysis of sugarcane bagasse: An approach on solid residues characterization. J. Supercrit. Fluids.

[13] Negro M.J., Manzanares P., Ballesteros I., Oliva J.M., Cabañas A., Ballesteros M., Davison B.H., Wyman C.E., Finkelstein M. (2003). Hydrothermal pretreatment conditions to enhance ethanol production from poplar biomass. Biotechnology for Fuels and Chemicals.

[14] Sakooei-Vayghan R., Peighambardoust S.H., Hesari J., Soltanzadeh M., Peressini D. (2020). Properties of Dried Apricots Pretreated by Ultrasound-Assisted Osmotic Dehydration and Application of Active Coatings. Food Technol. Biotechnol..

[15] Sakooei-Vayghan R., Peighambardoust S.H., Hesari J., Peressini D. (2020). Effects of osmotic dehydration (with and without sonication) and pectin-based coating pretreatments on functional properties and color of hot-air dried apricot cubes. Food Chem..

[16] Chen H.M., Fu X., Luo Z.G. (2015). Properties and extraction of pectin-enriched materials from sugar beet pulp by ultrasonic-assisted treatment combined with subcritical water. Food Chem..

[17] Gharibzahedi S.M.T., Smith B., Guo Y. (2019). Ultrasound-microwave assisted extraction of pectin from fig (*Ficus carica* L.) skin: Optimization, characterization and bioactivity. Carbohydr. Polym..

[18] Dehghani S., Peighambardoust S.H., Peighambardoust S.J., Fasihnia S.H., Khosrowshahi N.K., Gullón B., Lorenzo J.M. (2020). Optimization of the Amount of ZnO, CuO, and Ag Nanoparticles on Antibacterial Properties of Low-Density Polyethylene (LDPE) Films Using the Response Surface Method. Food Anal. Methods.

[19] Olmos J.C., Hansen M.E.Z. (2012). Enzymatic depolymerization of sugar beet pulp: Production and characterization of pectin and pectic-oligosaccharides as a potential source for functional carbohydrates. Chem. Eng. J..

[20] Huang X., Li D., Wang L. (2018). jun Effect of particle size of sugar beet pulp on the extraction and property of pectin. J. Food Eng..

[21] Muñoz-Almagro N., Valadez-Carmona L., Mendiola J.A., Ibáñez E., Villamiel M. (2019). Structural characterisation of pectin obtained from cacao pod husk. Comparison of conventional and subcritical water extraction. Carbohydr. Polym..

[22] Li D.Q., Jia X., Wei Z., Liu Z.Y. (2012). Box-Behnken experimental design for investigation of microwave-assisted extracted sugar beet pulp pectin. Carbohydr. Polym..

[23] Manrique G.D., Lajolo F.M. (2002). FT-IR spectroscopy as a tool for measuring degree of methyl esterification in pectins isolated from ripening papaya fruit. Postharvest Biol. Technol..

[24] Garna H., Mabon N., Wathelet B., Paquot M. (2004). New method for a two-step hydrolysis and chromatographic analysis of pectin neutral sugar chains. J. Agric. Food Chem..

[25] Jung J., Wicker L. (2012). Laccase mediated conjugation of sugar beet pectin and the effect on emulsion stability. Food Hydrocoll..

[26] Li W., Fan Z., Wu Y., Jiang Z., Shi R. (2019). Eco-friendly extraction and physicochemical properties of pectin from jackfruit peel waste with subcritical water. J. Sci. Food Agric..

[27] Brunner G. (2009). Near critical and supercritical water. Part I. Hydrolytic and hydrothermal processes. J. Supercrit. Fluids.

[28] Funami T., Nakauma M., Ishihara S., Tanaka R., Inoue T., Phillips G.O. (2011). Structural modifications of sugar beet pectin and the relationship of structure to functionality. Food Hydrocoll..

[29] Liew S.Q., Teoh W.H., Tan C.K., Yusoff R., Ngoh G.C. (2018). Subcritical water extraction of low methoxyl pectin from pomelo (*Citrus grandis* (L.) Osbeck) peels. Int. J. Biol. Macromol..

[30] Klinchongkon K., Khuwijitjaru P., Wiboonsirikul J., Adachi S. (2017). Extraction of Oligosaccharides from Passion Fruit Peel by Subcritical Water Treatment. J. Food Process Eng..

[31] Idrovo Encalada A.M., Pérez C.D., Gerschenson L.N., Rojas A.M., Fissore E.N., Encalada A.M.I., Pérez C.D., Gerschenson L.N., Rojas A.M., Fissore E.N. (2019). Gelling pectins from carrot leftovers extracted by industrial-enzymes with ultrasound pretreatment. LWT.

[32] Rombouts F.M., Thibault J.F. (1986). Feruloylated pectic substances from sugar-beet pulp. Carbohydr. Res..

[33] Pacheco M.T., Villamiel M., Moreno R., Moreno F.J. (2019). Structural and rheological properties of pectins extracted from industrial sugar beet by-products. Molecules.

[34] Min B., Lim J., Ko S., Lee K.G., Lee S.H., Lee S. (2011). Environmentally friendly preparation of pectins from agricultural byproducts and their structural/rheological characterization. Bioresour. Technol..

[35] Dartois A., Singh J., Kaur L., Singh H. (2010). Influence of Guar Gum on the in Vitro Starch Digestibility—Rheological and Microstructural Characteristics. Food Biophys..

[36] Gularte M.A., Rosell C.M. (2011). Physicochemical properties and enzymatic hydrolysis of different starches in the presence of hydrocolloids. Carbohydr. Polym..

[37] Kulicke W.-M., Eidam D., Kath F., Kix M., Kull A.H. (1996). Hydrocolloids and Rheology: Regulation of Visco-elastic Characteristics of Waxy Rice Starch in Mixtures with Galactomannans. Starch Stärke.

[38] Rosell C.M., Yokoyama W., Shoemaker C. (2011). Rheology of different hydrocolloids–rice starch blends. Effect of successive heating–cooling cycles. Carbohydr. Polym..

[39] Abioye V.F., Ade-Omowaye B.I.O., Babarinde G.O., Adesigbin M.K. (2011). Chemical, physico-chemical and sensory properties of soy-plantain flour. Afr. J. Food Sci..

[40] Biliaderis C.G., Maurice T.J., Vose J.R. (1980). Starch gelatinization phenomena studied by differential scanning calorimetry. J. Food Sci..

[41] Tester R.F., Sommerville M.D. (2003). The effects of non-starch polysaccharides on the extent of gelatinisation, swelling and α-amylase hydrolysis of maize and wheat starches. Food Hydrocoll..

[42] Krüger A., Ferrero C., Zaritzky N.E. (2003). Modelling corn starch swelling in batch systems: Effect of sucrose and hydrocolloids. J. Food Eng..

[43] (2006). AACC Approved Methods of the American Association of Cereal Chemist.

[44] Jafarzadeh-Moghaddam M., Shaddel R., Peighambardoust S.H. (2020). Sugar beet pectin extracted by ultrasound or conventional heating: A comparison. J. Food Sci. Technol..

[45] Gao J., Luo Z., Fu X., Luo F., Peng Z. (2012). Effect of enzymatic pretreatment on the synthesis and properties of phosphorylated amphoteric starch. Carbohydr. Polym..

[46] Aydar A. (2018). Utilization of Response Surface Methodology in Optimization of Extraction of Plant Materials. Statistical Approaches with Emphasis on Design of Experiments Applied to Chemical Processess.

[47] Boyacı İ.H. (2005). A new approach for determination of enzyme kinetic constants using response surface methodology. Biochem. Eng. J..

[48] Garleb K.A., Bourquin L.D., Fahey G.C. (1991). Galacturonate in Pectic Substances from Fruits and Vegetables: Comparison of Anion Exchange HPLC with Pulsed Amperometric Detection to Standard Colorimetric Procedure. J. Food Sci..

[49] Levigne S., Thomas M., Ralet M.C., Quemener B., Thibault J.F. (2002). Determination of the degrees of methylation and acetylation of pectins using a C18 column and internal standards. Food Hydrocoll..

[50] Voragen A.G.J., Schols H.A., Pilnik W. (1986). Determination of the degree of methylation and acetylation of pectins by h.p.l.c. Top. Catal..

